# Supplementary material for: Endoscopic Delivery of Polymers Reduces Delayed Bleeding after Gastric Endoscopic Submucosal Dissection: A Systematic Review and Meta-Analysis
Source: Polymers (Basel). 2022 Jun 13;14(12):2387. doi: 10.3390/polym14122387 (PMC9227627; doi:10.3390/polym14122387)
Supplement: Supplementary file 1 [file polymers-14-02387-s001.zip › Appendix SA Search Strategies.pdf]

Appendix A. Search strategies

The Medline search strategy was: ((hemorrhage) OR (haemorrhage) OR (Hemorrhages) OR (Bleeding) OR (blood loss)) AND ((stomach) OR (stomachs) OR (gastric)) AND ((Endoscopic Mucosal Resection) OR (Endoscopic Mucous Membrane Resection) OR (Endoscopic Submucosal Dissection)) AND ((prophylactic) OR (prophylaxis) OR (prevention) OR (prevent) OR (management) OR (hemostasis) OR (haemostasis) OR (hemostatic) OR (haemostatic) OR (closure) OR (tissue shielding) OR (endoclip) OR (endoclips) OR (clip) OR (clips) OR (clipping) OR (snare) OR (endoloop) OR (loop) OR (suture) OR (over the scope clip) OR (OTSC system) OR (OVESCO) OR (sutures) OR (Polyglycolic Acid) OR (fibrin glue)). Filters: **Journal Article, Humans, English** Sort by: **Most Recent**

The EMBASE search strategy was: ('hemorrhage'/exp OR hemorrhage OR 'haemorrhage'/exp OR haemorrhage OR hemorrhages OR 'bleeding'/exp OR bleeding OR (('blood'/exp OR blood) AND ('loss'/exp OR loss))) AND ('stomach'/exp OR stomach OR stomachs OR gastric) AND (endoscopic AND mucosal AND ('resection'/exp OR resection) OR (endoscopic AND mucous AND ('membrane'/exp OR membrane) AND ('resection'/exp OR resection)) OR (endoscopic AND submucosal AND ('dissection'/exp OR dissection))) AND (prophylactic OR 'prophylaxis'/exp OR prophylaxis OR 'prevention'/exp OR prevention OR prevent OR 'management'/exp OR management OR 'hemostasis'/exp OR hemostasis OR 'haemostasis'/exp

OR haemostasis OR hemostatic OR haemostatic OR closure OR (('tissue'/exp OR tissue) AND shielding) OR 'endoclip'/exp OR endoclip OR endoclips OR 'clip'/exp OR clip OR 'clips'/exp OR clips OR 'clipping'/exp OR clipping OR 'snare'/exp OR snare OR 'endoloop'/exp OR endoloop OR loop OR 'suture'/exp OR suture OR (over AND the AND ('scope'/exp OR scope) AND ('clip'/exp OR clip)) OR (('otsc'/exp OR otsc) AND system) OR ovesco OR 'sutures'/exp OR sutures OR (polyglycolic AND ('acid'/exp OR acid)) OR (('fibrin'/exp OR fibrin) AND ('glue'/exp OR glue))) AND **[article in press]/lim AND [humans]/lim AND [english]/lim AND [embase]/lim.**

The Cochrane Library search strategy was: (“Hemorrhage” [Mesh] OR (hemorrhage) OR (haemorrhage) OR (Hemorrhages) OR (Bleeding) OR (blood loss)) AND (“Endoscopic Mucosal Resection” [Mesh] OR (Endoscopic Mucosal Resection) OR (Endoscopic Mucous Membrane Resection) OR (Endoscopic Submucosal Dissection)) AND (“Stomach” [Mesh] OR (stomach) OR (stomachs) OR (gastric)) AND ((prophylactic) OR (prophylaxis) OR (prevention) OR (prevent) OR (management) OR (hemostasis) OR (haemostasis) OR (hemostatic) OR (haemostatic) OR (closure) OR (tissue shielding) OR (endoclip) OR (endoclips) OR (clip) OR (clips) OR (clipping) OR (snare) OR (endoloop) OR (loop) OR (suture) OR (over the scope clip) OR (OTSC system) OR (OVESCO) OR (sutures) OR (Polyglycolic Acid) OR (fibrin glue) OR “fibrin tissue adhesive” [Mesh] OR “Polyglycolic Acid” [Mesh] OR “Sutures” [Mesh]).
