# Supplementary material for: Endoscopic Delivery of Polymers Reduces Delayed Bleeding after Gastric Endoscopic Submucosal Dissection: A Systematic Review and Meta-Analysis
Source: Polymers (Basel). 2022 Jun 13;14(12):2387. doi: 10.3390/polym14122387 (PMC9227627; doi:10.3390/polym14122387)
Supplement: Supplementary file 1 [file polymers-14-02387-s001.zip › Supplementary Table S1 Quality of Studies.pdf]

Supplementary Table S1.1 Quality of cohort and single-arm studies

**Article (Author, year)**	**Selection**				**Comparability**	**Outcome**			**Total score**
	Representativeness of the exposed cohort	Selection of the nonexposed cohort	Ascertainment of exposure	Demonstration that outcome of interest was not present at star of study		Assessment of outcome	Length of follow - up	Adequacy of follow-up	Total score
Akimoto, 2022	1	0	1	1	0	1	1	1	6
Choi, 2008	1	1	1	1	2	1	0	1	8
Ego, 2020	1	1	1	1	2	1	1	1	9
Fukuda, 2016	1	1	1	1	2	1	1	1	9
Goto, 2020	1	0	1	1	0	1	0	1	5
Hahn, 2017	1	0	1	1	0	1	1	1	6
Han, 2020	1	0	1	1	0	1	1	1	6
Kawata, 2018	1	1	1	1	2	1	0	1	8
Kikuchi, 2019	1	1	1	1	2	1	1	1	9
Maekawa, 2015	1	0	1	1	0	1	1	0	5
Nishiyama,	1	0	1	1	0	1	0	1	5

2022									
Sabramaniam, 2019	1	0	1	1	0	1	1	1	6
Shiotsuki, 2021	1	1	1	1	2	1	1	1	9
Tan, 2016	1	1	1	1	2	1	1	1	9
Uraoka, 2016	1	0	1	1	0	1	1	1	6
Wang, 2020	1	1	1	1	2	1	1	1	9
Yoshida, 2021	1	0	1	1	0	1	1	0	5
Yu, 2022	1	1	1	1	2	1	0	0	7

Supplementary Table S1.2 Quality of non-randomized controlled studies

	**Bias due to confounding**	**Bias in selection of participants into the study**	**Bias in classification of interventions**	**Bias due to deviations from intended intervention**	**Bias due to missing data**	**Bias in measurement of outcomes**	**Bias in selection of the reported result**	**Overall bias**
Tsuji, 2015	Moderate risk	low risk	low risk	low risk	low risk	low risk	low risk	Moderate risk

Supplementary Table 1.3 Quality of randomized controlled studies

	**Random sequence**	**Allocation concealment**	**Blinding of participants and**	**Blinding of outcome**	**Incomplete outcome data**	**Selective reporting**	**Other bias**

	generation (selection bias)	(selection bias)	personnel (performance bias)	assessment (detection bias)	(attrition bias)	(reporting bias)	
Hwang, 2018	low risk	unclear risk	high risk	high risk	low risk	low risk	low risk
Jung, 2021	low risk	low risk	high risk	high risk	low risk	low risk	low risk
Kataoka, 2019	unclear risk	unclear risk	high risk	high risk	low risk	low risk	low risk
Lee, 2011	unclear risk	unclear risk	high risk	high risk	low risk	low risk	low risk
Mori, 2018	unclear risk	low risk	high risk	high risk	low risk	low risk	low risk
Zhang, 2013	unclear risk	unclear risk	high risk	high risk	low risk	low risk	low risk

Supplementary Table S1.4 Quality of case series

	**Selection**	**Ascertainment**		**Causality**				**Reporting**
	Representativeness	exposure	outcome	Ruled out alternative causes	Challenge/rechallenge phenomenon	Dose-response effect	Length of follow-up	
Abiko, 2021	1	1	1	0	-	-	0	1
Haddara, 2016	1	1	1	0	-	-	1	1
Goto, 2017	1	1	1	0	-	-	1	1
Kantsevoy,	1	1	1	0	-	-	1	1

2014								
Kobayashi, 2021	1	1	1	0	-	-	0	1
Pioche, 2016	1	1	1	0	-	-	1	1
