# Supplementary material for: Endoscopic Delivery of Polymers Reduces Delayed Bleeding after Gastric Endoscopic Submucosal Dissection: A Systematic Review and Meta-Analysis
Source: Polymers (Basel). 2022 Jun 13;14(12):2387. doi: 10.3390/polym14122387 (PMC9227627; doi:10.3390/polym14122387)
Supplement: Supplementary file 1 [file polymers-14-02387-s001.zip › Supplementary Table S2 network meta analysis results.pdf]

Supplementary Table S2. League table describing efficacy of all endoscopic approaches for prevention of delayed bleeding after gastric ESD according to network meta-analysis

In patients overall			
Control			
0.54 (-0.40, 1.56)	Endoscopic closure		
0.97 (0.13, 1.91)	0.42 (-0.88, 1.75)	Tissue shielding	
1.07 (0.28, 2.07)	0.52 (-0.73, 1.92)	0.10 (-1.11, 1.42)	Hemostatic spray
In high-risk patients			
Control			
0.41 (-0.49, 1.56)	Endoscopic closure		
1.15 (0.27, 2.13)	0.72 (-0.70, 2.05)	Tissue shielding	
0.36 (-0.95, 1.71)	-0.07 (-1.81, 1.54)	-0.79 (-2.43, 0.82)	Hemostatic spray
In low-risk patients			
Control			
0.23 (-4.02, 3.75)	Endoscopic closure		
0.33 (-4.21, 4.89)	0.11 (-5.50, 6.44)	Tissue shielding	
2.25 (0.13, 5.18)	2.06 (-1.92, 7.45)	1.89 (-2.83, 7.49)	Hemostatic spray
