# Supplementary figures and images for: Endoscopic Delivery of Polymers Reduces Delayed Bleeding after Gastric Endoscopic Submucosal Dissection: A Systematic Review and Meta-Analysis
Source: Polymers (Basel). 2022 Jun 13;14(12):2387. doi: 10.3390/polym14122387 (PMC9227627; doi:10.3390/polym14122387)

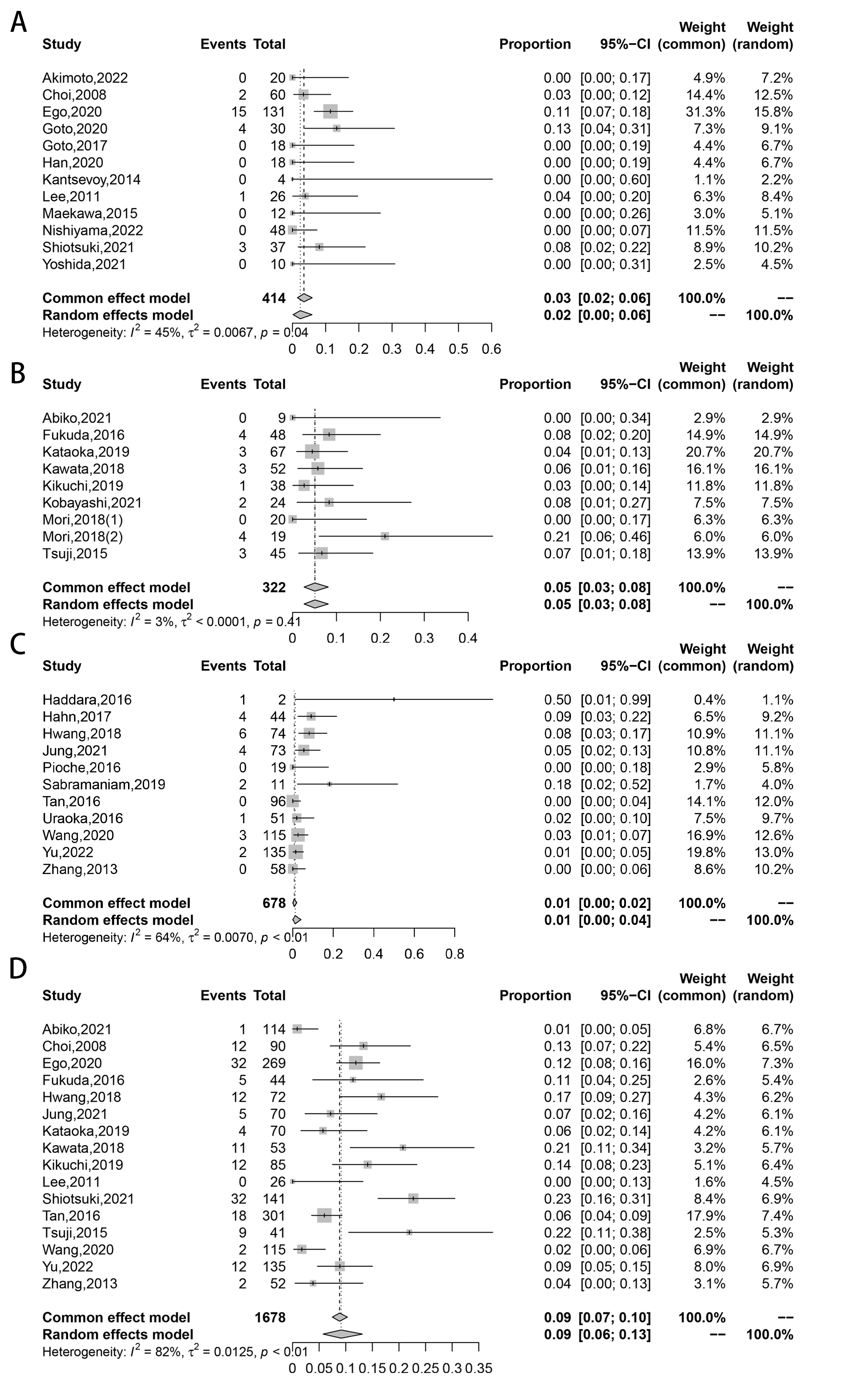

Supplement: Supplementary file 1 [file polymers-14-02387-s001.zip › Supplementary Figure S1 non-comparative trial synthesis of delayed-bleeding rates.tif]

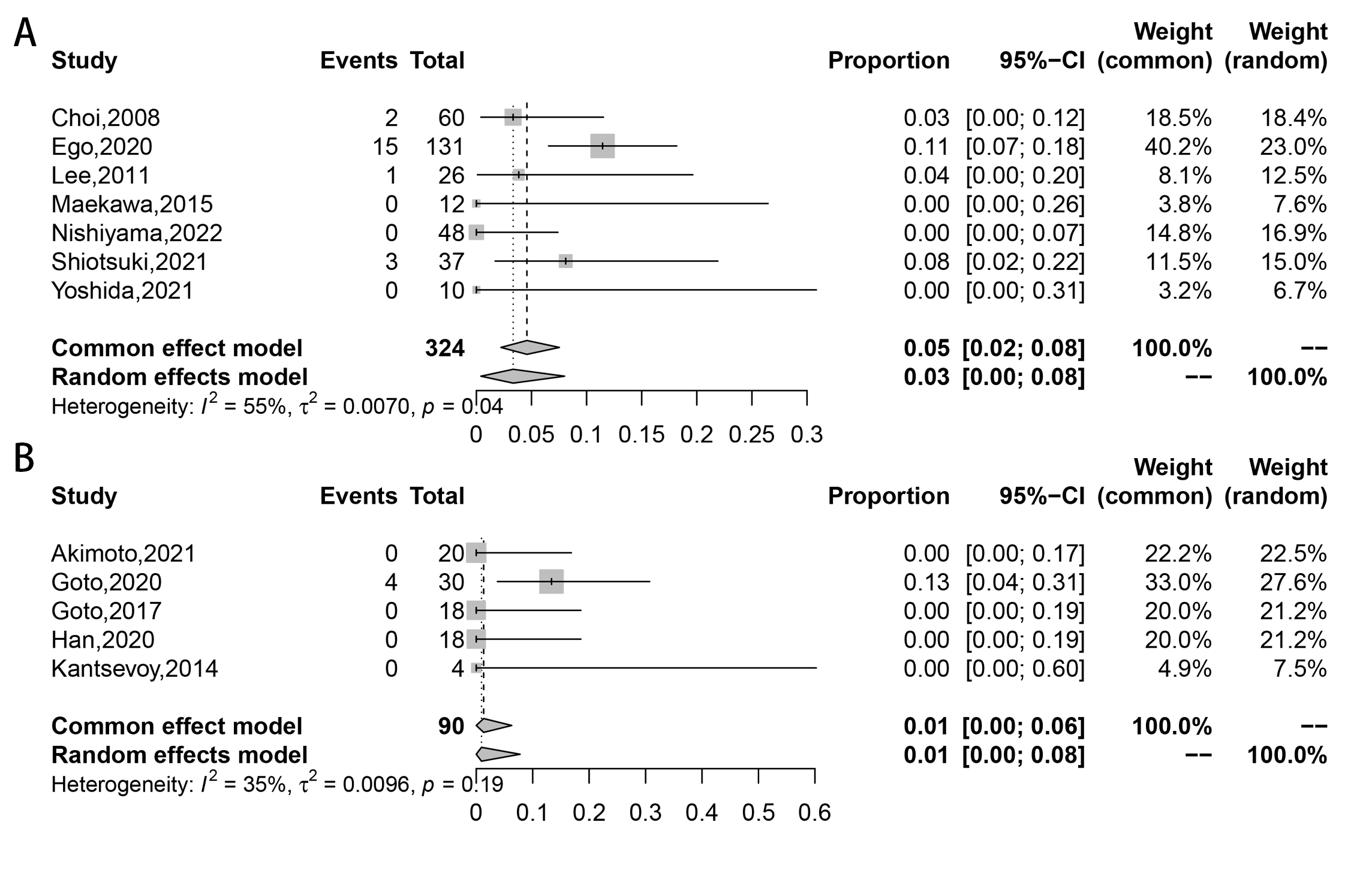

Supplement: Supplementary file 1 [file polymers-14-02387-s001.zip › Supplementary Figure S2 non-comparative trial synthesis of delayed-bleeding rates in endoscopic closure methods.tif]

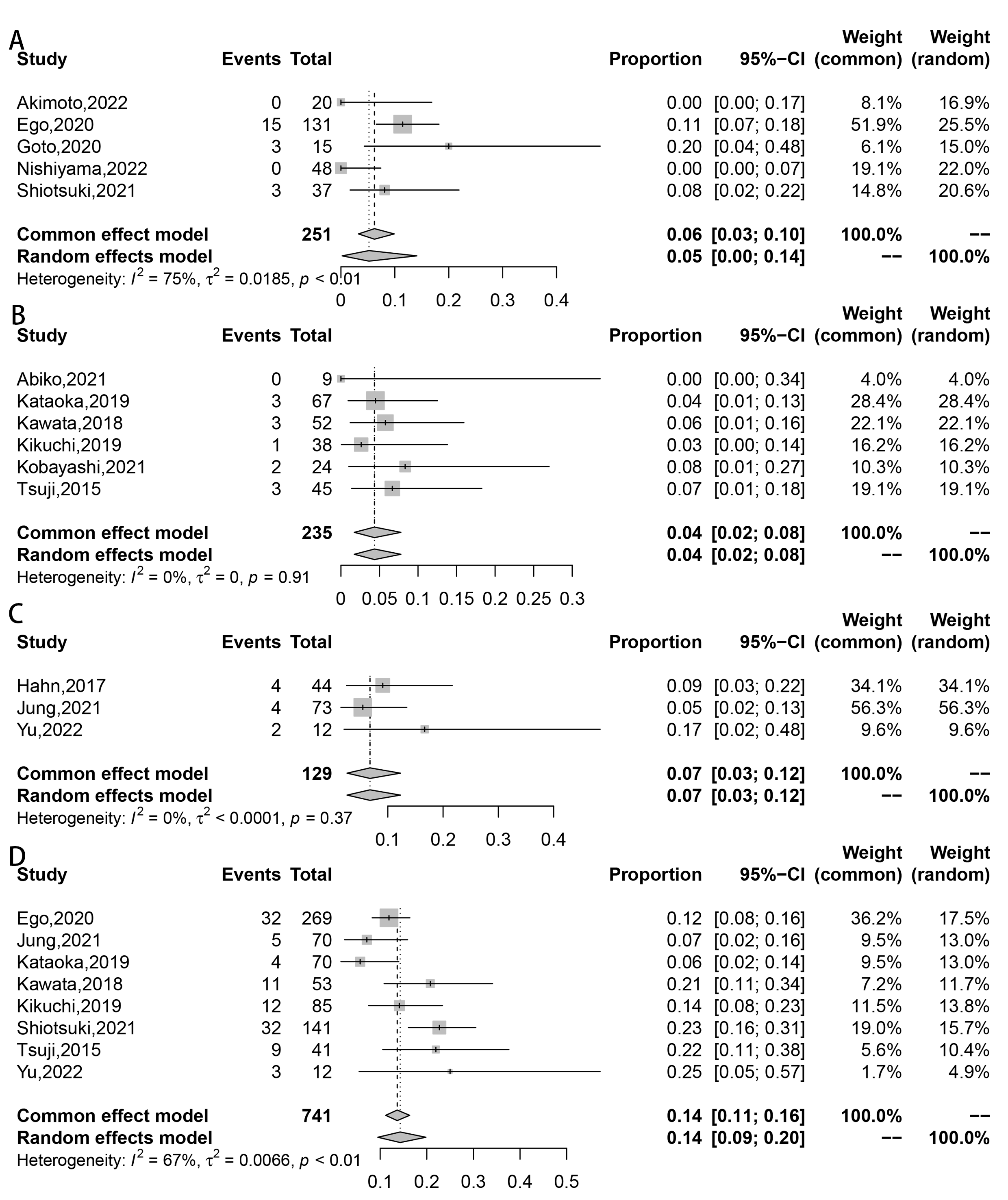

Supplement: Supplementary file 1 [file polymers-14-02387-s001.zip › Supplementary Figure S3 non-comparative trial synthesis of delayed bleeding rates in high-risk patients.tif]

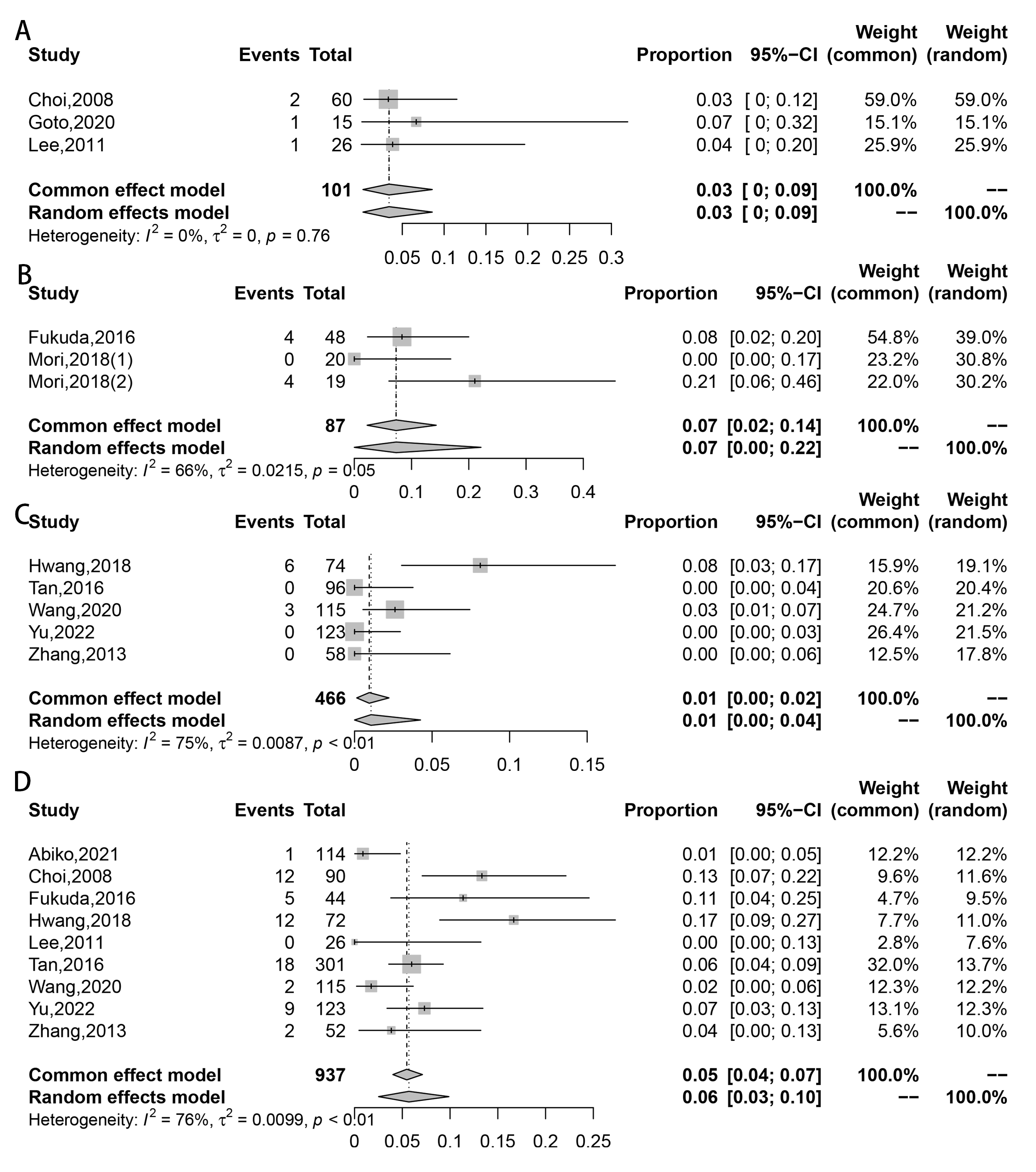

Supplement: Supplementary file 1 [file polymers-14-02387-s001.zip › Supplementary Figure S4 non-comparative trial synthesis of delayed bleeding rates in low-risk patients.tif]

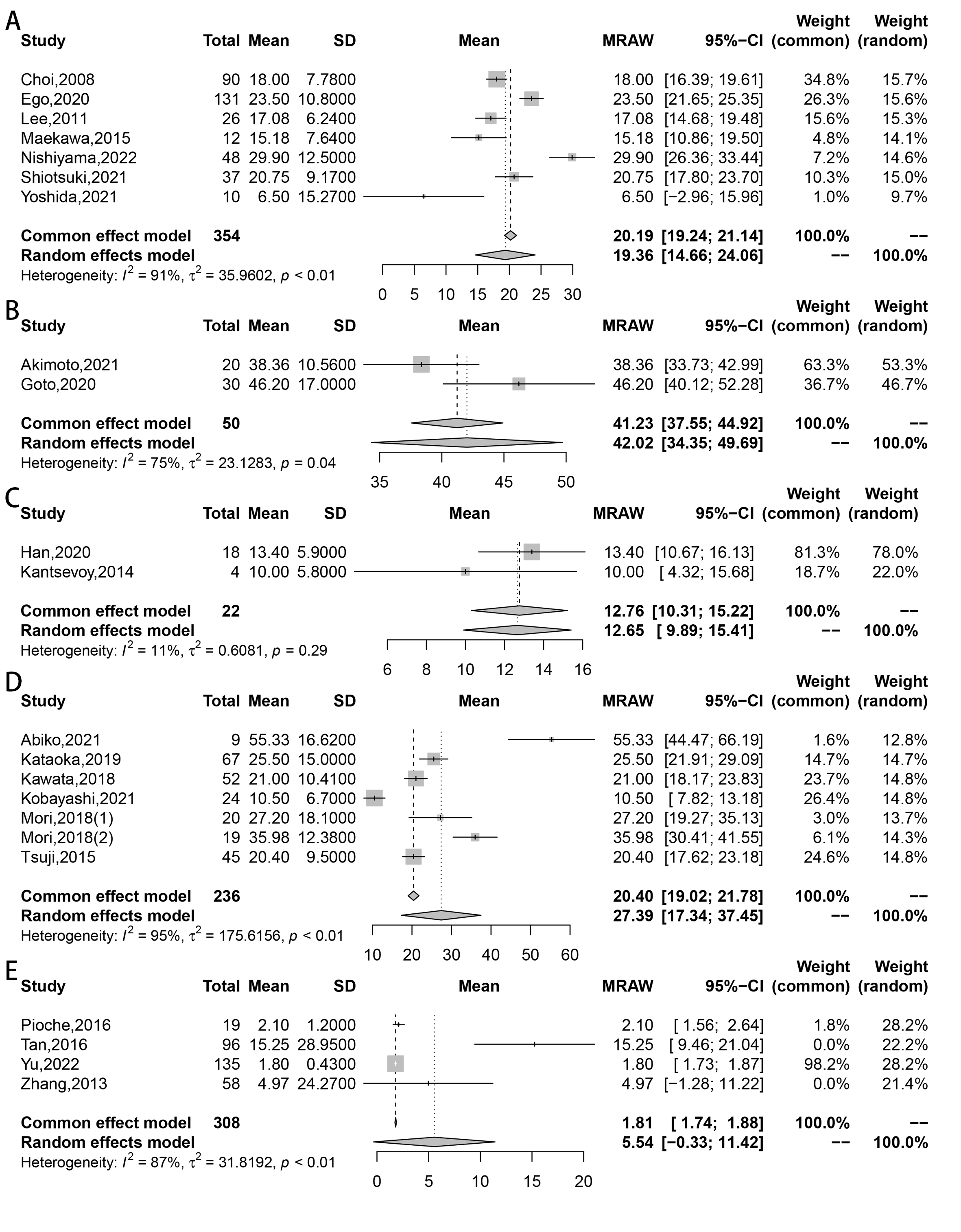

Supplement: Supplementary file 1 [file polymers-14-02387-s001.zip › Supplementary Figure S5 non-comparative trial synthesis of procedure time.tif]

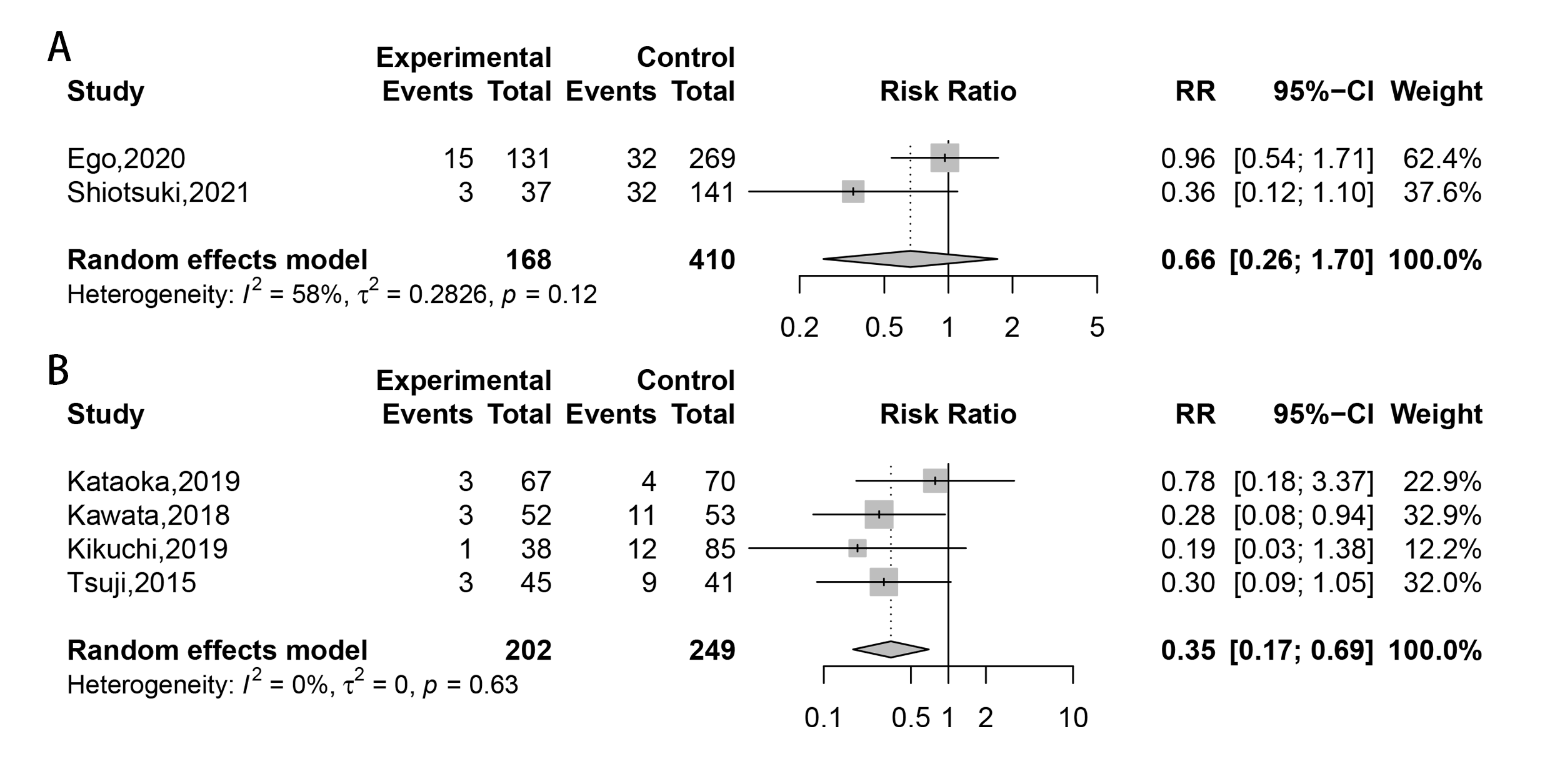

Supplement: Supplementary file 1 [file polymers-14-02387-s001.zip › Supplementary Figure S6 pairwise meta analysis in high-risk patients.tif]

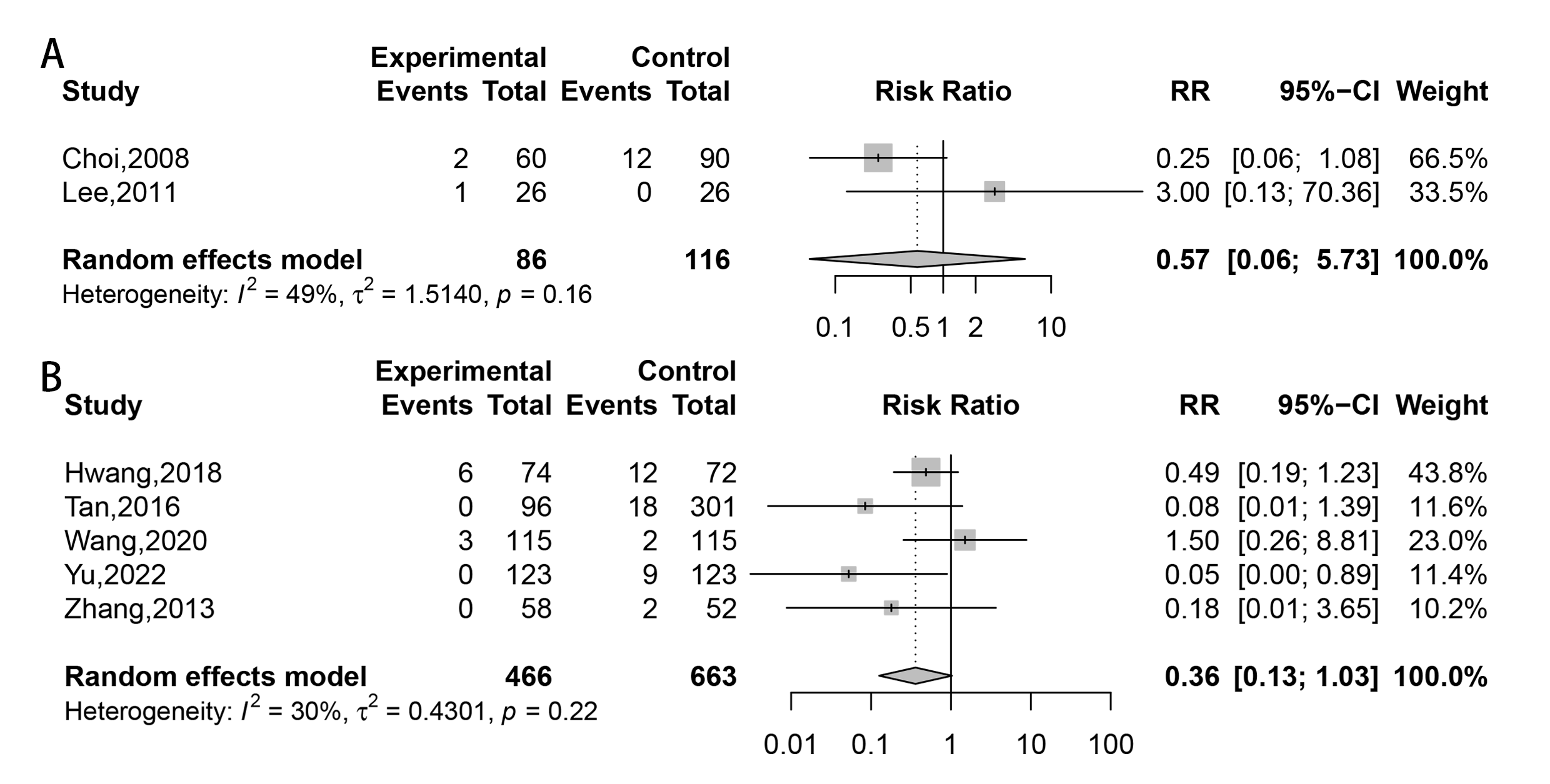

Supplement: Supplementary file 1 [file polymers-14-02387-s001.zip › Supplementary Figure S7 pairwise meta analysis in low-risk patients.tif]

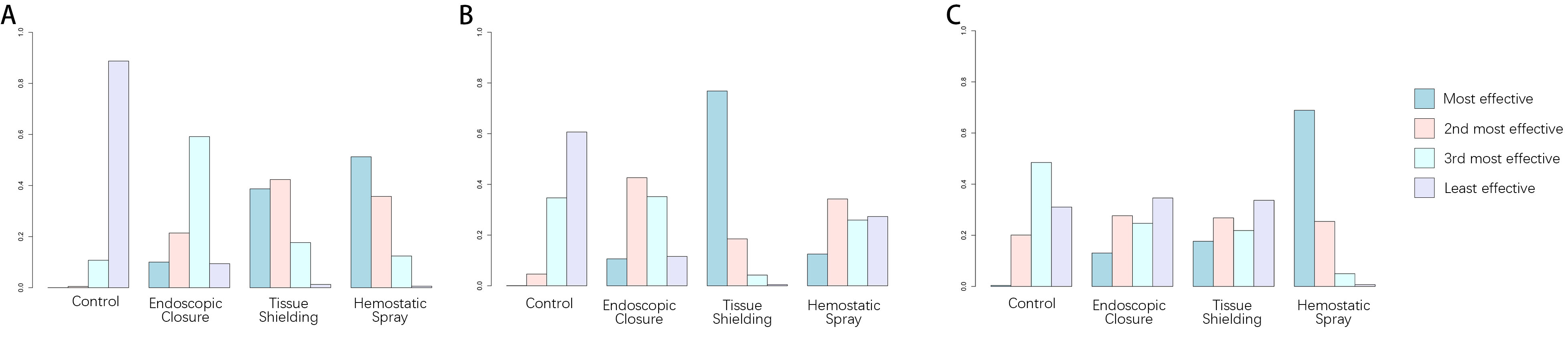

Supplement: Supplementary file 1 [file polymers-14-02387-s001.zip › Supplementary Figure S8 Ranking.tif]

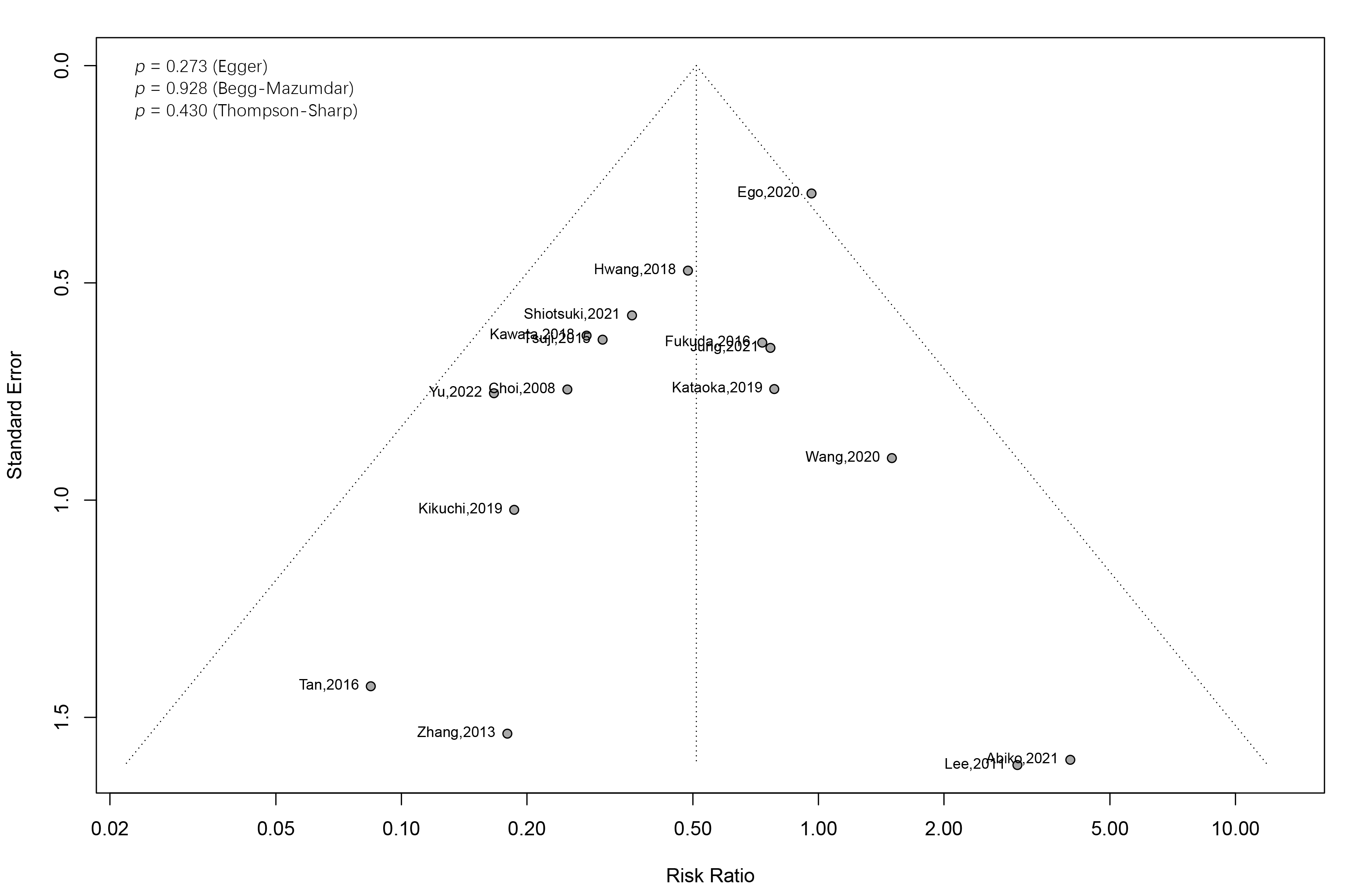

Supplement: Supplementary file 1 [file polymers-14-02387-s001.zip › Supplementary Figure S9 Publication bias.tif]
